# Varicella-zoster-virus vaccination in immunosuppressed children with rheumatic diseases using a pre-vaccination check list

**DOI:** 10.1186/s12969-018-0231-3

**Published:** 2018-03-02

**Authors:** Fabian Speth, Claas H. Hinze, Susanne Andel, Thomas Mertens, Johannes-Peter Haas

**Affiliations:** 1 German Center for Pediatric and Adolescent Rheumatology, Gehfeldstr. 24, 82467 Garmisch-Partenkirchen, Germany; 2 Department of Pediatric Rheumatology and Immunology, University Hospital Münster, Albert-Schweitzer-Campus I, Building W30, 48149 Münster, Germany; 3 Institute of Virology, Ulm University Medical Center, Albert-Einstein-Allee 11, 89081 Ulm, Germany

**Keywords:** Biologic agents, DMARDs, Immunologic tests, Juvenile chronic arthritis, Pediatric rheumatic diseases, Varicella zoster virus, Vaccination

## Abstract

**Background:**

The goal of this study was to apply the varicella zoster virus (VZV) vaccine to patients with pediatric rheumatic diseases (PRD) at risk for severe chickenpox, without interrupting their current immunosuppression, including biological agents, using an immunological-based pre-vaccination checklist to assure safety. A pre-vaccination checklist was implemented to ensure adequate immune competence prior to immunization.

**Methods:**

This prospective study included seronegative patients (VZV-IgG ≤200 mIU/ml) and patients who had previously received only a single dose of VZV vaccine. All vaccinees demonstrated clinically inactive PRD. Patients were categorized according to their actual treatment in low-intensity IS (LIIS) and high-intensity IS (HIIS) including biological therapy. The pre-vaccination checklist defined thresholds for the following basic laboratory tests: white blood cell count ≥3000/mm^3^, lymphocytes ≥1200/mm^3^, serum IgG ≥500 mg/dl, IgM ≥20 mg/dl, tetanus toxoid antibody ≥0.1 IU/ml. In case of HIIS additional specifications included a CD4+ lymphocyte count ≥200/mm^3^ and a positive T-cell function (via analyzable positive control of a standard tuberculosis interferon-gamma-release-assay (TB-IGRA) indicating mitogen-induced T cell proliferation). Patients who met the criteria of the pre-vaccination checklist received the first and/or second VZV vaccination. Immunologic response and side effects were monitored.

**Results:**

Twenty-three patients were recruited of whom nine had already received one VZV immunization before initiating IS. All patients met the pre-vaccination checklist criteria despite ongoing IS. There was no overall difference in VZV-IgG levels when comparing the LIIS (*n*=9) and HIIS (*n*=14) groups. In total, 21 patients (91%) showed a positive vaccination response, after the first immunization the median VZV-IgG across all patients was 224 (59-1219) mIU/ml (median (range)), after booster immunization it increased to 882 (30-4685) mIU/ml. Two patients in the HIIS group failed to raise positive VZV-IgG, despite booster immunization. All nine patients receiving only the second immunization on IS reached high titers of VZV-IgG >500 mIU/ml (1117 (513-4685) mIU/ml). There were no cases of rash or other vaccine-induced varicella disease symptoms and no evidence of PRD flare.

**Conclusions:**

VZV vaccination is safe and largely immunogenic in children with ongoing IS fulfilling an immunological based pre-vaccination checklist. This new approach is based on immunologic function rather than on type of medications.

**Trial registration number:**

ISRCRTN trial registration number 21654693, date of registration February 12, 2018, retrospectively registered.

**Electronic supplementary material:**

The online version of this article (10.1186/s12969-018-0231-3) contains supplementary material, which is available to authorized users.

## Background

Varicella-zoster virus (VZV) the etiologic agent of chickenpox and herpes zoster [HZ], is highly contagious and still endemic worldwide. Immunosuppressed individuals are at substantial risk for severe disease courses [1]. Therefore, the primary motivation for the development of the live attenuated VZV vaccine in the 1970s was to use it as a targeted vaccine to protect vulnerable children during cancer chemotherapy [2, 3]. The safety of administering the VZV vaccine has been studied extensively in immunosuppressed children with malignant and non-malignant disease, e.g. following kidney, liver or intestinal transplantation, nephrotic syndrome or inflammatory bowel disease, demonstrating marked safety and good efficacy [2–10]. However, in cases of extreme T cell deficiency with CD4+ T cell count <100/mm^3^ in the context of severe combined immunodeficiency and the acquired immunodeficiency syndrome, severe chickenpox induced by the VZV vaccine strain was observed [11, 12]. In trials of VZV vaccination in immunosuppressed children, immunological testing was heterogeneous and, for example, included ruling out severe lymphopenia, and the demonstration of preserved immunoreactivity by measurement of total IgG level, antibody titers to inactivated vaccines, CD4+ T cell counts and intracutaneous or in vitro T-cell function tests [2, 6, 7, 9, 10, 13]. Smaller trials focused on children with pediatric rheumatic diseases (PRD) such as systemic lupus erythematosus and juvenile idiopathic arthritis (JIA) [14–16]. Children within these trials were treated with glucocorticoids (prednisone up to 0.7 mg/kg body weight), methotrexate, leflunomide, azathioprine, 6-mercaptopurine, cyclosporine, tacrolimus, and, in individual cases, biologics. Again, there were no relevant adverse effects and efficacy, as measured by the prevention of breakthrough VZV-associated disease and an increase in VZV-IgG level, was good. However, the VZV-IgG response appeared slightly diminished when compared to healthy controls in one study [15].

The current recommendations for vaccination of children with rheumatic disease by the European League Against Rheumatism (EULAR) state not to administer live-virus vaccines to patients on therapy with high-dose disease-modifying antirheumatic drugs (DMARD), high-dose glucocorticoids or biological agents except on a case-to case basis [17]. However, guidance on how to decide on a case-to-case basis is not offered.

The goal of this study was to apply the VZV vaccine to patients with PRD at risk for severe chickenpox, without interrupting their current IS, including biological agents, using an immunological-based pre-vaccination checklist to assure safety. The checklist contained only easy-to-obtain clinical and immunologic parameters and could be used for the whole spectrum of currently used/ standard IS. This new approach is based on immunologic function rather than on type of medications. Patients demonstrating immunoreactivity despite IS received live vaccine and safety and immunogenicity data were obtained.

## Methods

### Participants and stratification method

A prospective single-center study was conducted at the German Center for Pediatric and Adolescent Rheumatology, Garmisch-Partenkirchen (ISRCRTN: 21654693, retrospectively registered). The study was approved by the Institutional Board of Ethics in Medical Research of the Bavarian Chamber of Physicians. Written informed consent was obtained from all parents and the patients. Within the inclusion period from April 2012 to Mai 2013, 2802 patients between the ages of 2 to 17 years were screened by reviewing their immunization records, a questionnaire asking about prior chickenpox or herpes zoster and, in case of absence of chickenpox, herpes zoster and vaccination, by VZV-IgG screening. Inclusion criteria for the intervention part of the study were: (1) negative medical history for chickenpox and herpes zoster, (2) ≤ 1 prior dose of the VZV vaccine, (3) in case of first VZV vaccination, laboratory evidence of susceptibility for chickenpox defined as VZV-IgG either classified as negative (<160 mIU/ml) or equivocal (160-200 mIU/ml) (test system: VZV-IgG-ELISA (medac GmbH, Wedel) calibrated based on WHO reference preparation allowing quantitative measurements), (4) diagnosis of an inflammatory PRD, (5) clinically inactive disease as defined by the American College of Rheumatology (ACR) criteria in case of JIA [18] or a physician’s global score of <1 in case of other PRDs, and (6) no change of IS for at least 3 months prior to the vaccination. Exclusion criteria were as follows: (1) acute febrile disease, (2) current clinical or laboratory evidence for lack of immunologic reactivity (see pre-vaccination checklist below), (3) known hypersensitivity to constituents of the varicella vaccine, (4) measles, mumps, rubella (MMR) vaccination within 4 weeks prior to VZV vaccination, (5) treatment with IS other than those mentioned in the pre-vaccination checklist, i.v. glucocorticoid pulse therapy or a prednisone-equivalent dose of ≥2mg/kg/day or ≥20mg/day for > 2 weeks within less than 4 weeks prior to vaccination, cyclophosphamide pulse <6 months ago, rituximab without B-cell reconstitution, intravenous immune globulins (IVIG) <6 months ago (high-dose IVIG (2g/kg) <11 months), therapy with aspirin until 6 week post-vaccination or vi) any blood products <3 months prior to vaccination.

### Definition of low-intensity IS (LIIS) and high-intensity IS (HIIS)

After consultation with the German Standing Committee on Vaccination (STIKO), we graded the intensity of the IS by applying laboratory testing to determine the current immunologic reactivity that should allow control of the OKA vaccine strain. Definitions regarding the level of IS were based on available recommendations [17, 19]. Medications for which no previous grading or sufficient experience regarding live virus vaccination existed, such as biologic DMARDs, mycophenolate mofetil and mechanistic Target of Rapamycin (mTOR) inhibitors, e.g. sirolimus and everolimus, were considered to represent HIIS.

- LIIS included: (1) methotrexate (MTX) ≤15mg/m^2^/week or max. 15mg/week, (2) prednisolone (PDN) ≤0.5mg/kg/day (max. 10 mg/day) or (3) azathioprine (AZA) ≤2mg/kg/day (max. 100mg/day).
- HIIS included: (1) MTX >15mg/m^2^/week or >15mg/week, (2) PDN >0.5 to <2mg/kg/day or >10 to <20mg/day, (3) AZA >2 to 3mg/kg/day, (4) leflunomide ≤0,5mg/kg/days or ≤20mg/day, (5) cyclosporine A ≤ 3mg/kg/day with trough level ≤100μg/l, (6) tacrolimus and mTOR inhibitors with trough level ≤4ng/ml, (7) mycophenolate mofetil (MMF) ≤1200mg/m^2^/day or up to 2g/day, (7) etanercept ≤0.8mg/kg/week or up to 50mg/week, (8) adalimumab ≤24mg/m^2^ or up to 40mg every 2 weeks, (9) infliximab ≤6mg/kg up to every 4 weeks, (10) tocilizumab ≤12mg/kg up to every 2 weeks (if body weight ≤30kg) or ≤8mg/kg up to every 2 weeks if body weight ≥30kg, (11) anakinra ≤3mg/kg/day up to 150mg/day, and (12) abatacept ≤10mg/kg every 4 weeks. Combination therapy was allowed except for combination of two biologic DMARDs.

### Pre-vaccination checklist

A checklist was developed, consisting of items regarding medical history, physical examination, contraindications, grading the level of IS and basic laboratory examinations for all patients (Table 1). Basic laboratory testing included white blood cells (WBC) and lymphocyte count, serum IgG and IgM levels and tetanus toxoid antibody level. Cut-off levels were WBC ≥3000/mm^3^, lymphocytes ≥1200/mm^3^, serum IgG ≥500 mg/dl, IgM ≥20 mg/dl, tetanus toxoid antibody ≥0.1 IU/ml [20]. If tetanus antibody-level was <0.1 IU/ml, tetanus (booster) vaccination was administered and antibody testing repeated 4 weeks later. Additional (extended) laboratory examinations were pursued for patients receiving HIIS or patients on LIIS with abnormal basic laboratory test results and included the following parameters and cut-off levels: normal CD4+ T cell count (cut-off ≥200/mm^3^ if age >5 years or ≥500/mm^3^ if age 2-5 years) and a positive T cell function test (CMI). As an easy-to-obtain T cell function test we used a commercial interferon-gamma release assay (IGRA), the TB-EliSpot® test, which contains a mitogen as positive control (*phytohemagglutinin*). Patients exceeding the respective cut-off levels had adequate immunologic reactivity for the purpose of this study and VZV vaccination was offered.

**Table 1:** Checklist regarding clinical and immunological requirements prior to varicella zoster virus vaccination

Step 1: Rating of immunosuppressive therapy	Step 2: Prerequisites	Step 3: Testing protocol	Step 4: Decision about vaccination
Low-intensity immunosuppression (LIIS) □ Prednisone ≤ 0,5mg/kg (max. 10mg) daily □ Methotrexate ≤ 15mg/m^2^ (max. 15mg) weekly □ Azathioprine ≤ 2mg/kg (max. 100mg) daily High-intensity immunosuppression (HIIS) *Glucocorticoids and synthetic DMARDs* □ Prednisone > 0,5 to < 2mg/kg (> 10mg to < 20mg) daily □ Methotrexate > 15mg/m^2^ or > 15mg to 30mg weekly □ Azathioprine > 2 to 3mg/kg daily □ Leflunomide ≤ 0,5mg/kg (max. 20mg) daily □ Cyclosporine ≤ 3mg/kg daily (trough level ≤ 100μg/l) □ mTOR-inhibitors (sirolimus or everolimus) (trough level ≤ 4 μg/l) □ Mycophenolate ≤ 1200mg/m2 (max. 2g) daily *Biological DMARDs* □ Abatacept ≤ 10mg/kg per 4 weeks □ Adalimumab ≤ 24mg/m^2^ (max. 40mg) per 2 weeks □ Anakinra ≤ 3mg/kg (max. 150mg) daily □ Etanercept ≤ 0,8mg/kg (max. 50mg) weekly □ Infliximab ≤ 6mg/kg per 4 weeks □ Tocilizumab ≤ 12mg/kg biweekly if weight < 30kg □ Tocilizumab ≤ 8mg/kg biweekly if weight > 30kg Special situations: • If on canakinumab, consider switching to anakinra temporarily.	I. Medical history and physical exam □ No family history or clinical evidence of primary immunodeficiency □ No measles-mumps-rubella vaccination at the same time or within < 4 weeks before VV □ For 2nd VV: At least 3 months from first dose of VV for patients on HIIS II. Clinical assessment □ Inactive rheumatic disease □ Stable antirheumatic therapy for at least 3 months □ No active infection III. Medications □ Not more than 2 synthetic DMARDs □ Not more than 1 biological agent +/− 1 synthetic DMARD □ No IV methylprednisolone pulse therapy or oral prednisone ≥ 2mg/kg or ≥ 20mg daily for > 2 weeks within < 1 month before and after VV □ No IV cyclophosphamide in previous 6 months □ No rituximab in previous 6 months or lasting B-cell deficiency □ No IVIG in previous 6 months (high-dose IVIG [2g/kg] 11 months) □ No blood products in previous 3 months □ No therapy with aspirin planned in 6 weeks after vaccination	Basic laboratory testing for all patients: □ VZV-IgG < 200 mIU/ml (prior to1st VV) □ WBC ≥ 3,000/mm^3^ □ Lymphocytes ≥ 1,200/mm^3^ □ IgG ≥ 500 mg/dl □ IgM ≥ 20mg/dl □ Tetanus toxoid antibody ≥ 0.1 IU/ml Additional laboratory testing for patients on HIIS or LIIS with abnormal basic lab result: □ CD4 cell count > 200/mm^3^ if > 5 yrs old OR CD4 cell count > 500/mm^3^ if 1-5 yrs old □ T cell function testing^a^ positive Special situations: • If WBC < 3000/mm^3^: • exclude neutropenia <1500/mm^3^ • If lymphocytes 700-1.200/mm^3^: • rule out low CD4+ T-cells • If Lymphocytes < 700/mm^3^: • absolute contraindication • If IgG < 500mg/dl or IgM < 20mg/dl: • rule out humoral and cellular immunodeficiency • If tetanus antibody-level < 0,10IU/ml: • give tetanus vaccination and control serologic response in 4 weeks	□ All prerequisites are met □ Basic laboratory testing normal □ Additional lab testing normal (if applicable), or additional testing not applicable IF ALL THREE ITEMS ABOVE ARE CHECKED, VV IS SUPPORTED WITHOUT SUSPENSION OF THE CURRENT IMMUNOSUPPRESSION.^b,c^

Abbreviations: *DMARD* disease-modifying antirheumatic drugs, *HIIS* high-intensity immunosuppression, *LIIS* low-intensity immunosuppression, *VV* varicella vaccination, *VZV-IgG* anti varicella zoster virus titre, *WBC* white blood cell count.

^a^via Tuberculosis Interferon-gamma-release assays (for example, TB-EliSpot^®^ or Quantiferon^®^ test) demonstrating a positive (control) mitogen response OR other positive dedicated T cell function testing.

^b^Patientsfullfilling these pre-vaccination criteria, also meet the immunological precautions requested by the manufacturer of Varilrix (Glaxo-Smith-Kline) for the in-label application.

^c^Especially in case of breakthrough or vaccine-induced VZV disease with >50 skin lesions or a rash lasting >7 days: strongly consider treatment with acyclovir and contact pediatric rheumatologist for a possible reduction of immunosuppressive therapy

### Vaccination

Patients stratified to fullfill the pre-vaccination criteria, also met the immunological precautions requested by the manufacturer of Varilrix® (Glaxo-Smith-Kline) for the in-label application of the VZV vaccine on IS (absolute lymphocyte count >1200/mm^3^ and/or adequate CMI). Varilrix® contains the live attenuated Oka strain at a concentration of at least 10^3.3^ plaque-forming units per 0.5ml. Participants received a standard dose of the vaccine 0.5ml subcutaneously. A second dose was given at an interval of at least 6 weeks on LIIS and 3 months on HIIS [19]. Patients, parents and the primary care pediatrician received a written instruction to initiate treatment with acyclovir in case of VZV disease with >50 skin lesions or a rash lasting >7 days and to contact their pediatric rheumatologist to decide on A reduction of the IS.

### Determination of safety

Patients/parents and pediatricians completed a separate questionnaire between 4-12 weeks following vaccination asking for vaccination-associated side effects or signs for flare of the PRD. If any sign of side effect was present, further details were obtained by telephone interview.

### Determination of immunogenicity and efficacy

VZV-IgG response was measured using a blood sample drawn between 4 to 12 weeks after vaccination. Furthermore, patients were interviewed after a longer interval (median 3 years) to determine if post-vaccination contact to VZV and/or breakthrough chickenpox or HZ had occurred.

### Statistical analysis

Statistical analysis was performed using GraphPad Prism 6 (GraphPad Software, La Jolla, CA, USA) and Microsoft Excel 2010 (Microsoft, Redmond, WA, USA). Demographic and baseline laboratory characteristics were described using medians and range. Differences between patients receiving LIIS and those receiving HIIS were estimated with the chi-squared test for proportions and with the Mann-Whitney test for interval variables.

## Results

### Screening for lack of varicella immunity and recruitment

**Fig. 1:**
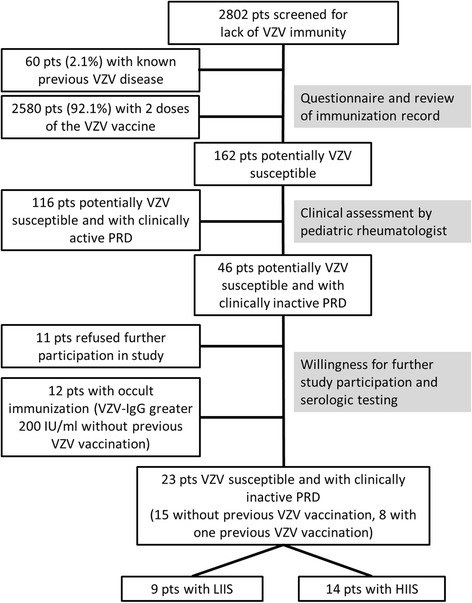
Patient disposition. *Abbreviations: HIIS* high-intensity immunosuppression, *LIIS* low-intensity immunosuppression, *VZV* Varicella-zoster-virus

Within a period of 13 months 2802 patients with PRD were screened for their VZV-susceptibility by a questionnaire. Sixty patients (2.1%) reported a history of chickenpox while 2580 patients (92.1%) had received two doses of the VZV vaccine as recommended in Germany by the STIKO. Of the remaining 162 patients (5,8%) potentially susceptible for VZV, 116 had clinically active PRD and 46 had clinically inactive disease. Of 46 patients with clinically inactive disease, 11 refused further study participation. Of the 35 potentially VZV susceptible patients willing to further participate in the study, 12 had a VZV-IgG >200 IU/ml indicating prior contact to VZV. Additionally, we observed titers classified as negative (<160 mIU/ml) in 4 out of 9 patients that had received a single VZV vaccination prior to the study (median 230 mIU/ml, range 57-1003 mIU/ml). Twenty-three patients entered the interventional part of this study (Fig. 1).

### Participants and baseline laboratory and immunologic data

Demographic data, disease type, current treatments and baseline laboratory test results of the patients vaccinated in the study are shown in Table 2. One patient had a low tetanus toxoid antibody level and received a successful tetanus booster-vaccination. All other parameters were above the predefined thresholds in all patients.

**Table 2:** Demographic characteristics and baseline immunological data for participants

Characteristic	Overall	Low-intensity immunosuppression (LIIS)	High-intensity immunosuppression (HIIS)	*p* value
Number	23	9 (39%)	14 (61%)	
Female	17 (74%)	8 (89%)	10 (71%)	0.32^a^
Age, median (range) years	9.6 (1.8-17.8)	8.3 (1.8-17.8)	9.7 (2.7-17.8)	0.33^b^
Disease type		8 JIA	11 JIA	
		6 oligoarthritis	3 oligoarthritis	
		1 polyarthritis	3 polyarthritis	
		1 psoriatic arthritis	4 systemic arthritis	
		1 Sjögren syndrome	1 psoriatic arthritis	
			2 JDM	
			1 MPA	
Immunosuppressive drug therapy		9 MTX <15mg/m^2^/wk	2 MTX ≥15mg/m^2^/wk	N/A
			1 MTX+TCZ	
			1 MTX+ADA	
			1 MTX+ANK+PDN	
			1 LEF	
			1 LEF+ABA	
			1 LEF+ANK+PDN	
			1 LEF+ETN+PDN	
			1 LEF+TCZ	
			2 ETN	
			1 ETN+PDN	
			1 MMF	
Varicella vaccine history				0.31^a^
0 previous doses	15	7	8	
1 previous dose	8	2	6	
Baseline white blood cell count, median (range) per mm^3^	5900 (4100-8311)	5100 (4100-6900)	6715 (4400-8311)	0.12^b^
Baseline absolute lymphocyte count, median (range) per mm^3^	2433 (1156-4647)	2295 (1156-3200)	2519 (1158-4647)	0.63^b^
Serum IgG, mean (SD) mg/dl	793 (542-1803)	793 (637-1803)	787 (542-1403)	0.61^b^
Tetanus toxoid antibody, mean (SD) IU/ml	1.4 (0.1-6.2)	2.2 (0.16-6.2)	0.6 (0.1-2.7)	0.01^b^
CD4+ T cell count, mean (SD) per mm^3^	N/A	N/A	1371 (546)	N/A
Positive T cell function, n (%)	N/A	N/A	14 (100)	N/A

Abbreviations: *ABA* abatacept, *ADA* adalimumab, *ANK* anakinra, *JIA* juvenile idiopathic arthritis, *JDM* juvenile dermatomyositis, *ETN* etanercept, *LEF* leflunomide, *MMF* mycophenolate mofetil, *MPA* microscopic polyangiitis, *MTX* methotrexate, *N/A* not applicable, *PDN* prednisolone, *SD* standard deviation, *TCZ* tocilizumab, *WBC* white blood cells.

^a^chi-square test; ^b^Mann Whitney test

### Administration of the varicella vaccine

Nine patients who had previously received one dose of the VZV vaccine received a second dose in the context of this study (2 in the LIIS group, 7 in the HIIS group). Of the 15 patients naïve for VZV, six only received one vaccination (3 in the LIIS group, 3 in the HIIS group) while nine received two (4 in the LIIS group, 5 in the HIIS group). Among the six patients who received only one vaccination, one patient refused a second vaccination, and five patients (and their local pediatricians) deferred a second vaccination due to an increase in VZV-IgG-level after just one vaccination. We suggested to repeat VZV-IgG level measurements in these instances and to offer the booster vaccine in case of a negative or borderline VZV-IgG level.

### VZV-IgG response following vaccination

**Fig. 2:**
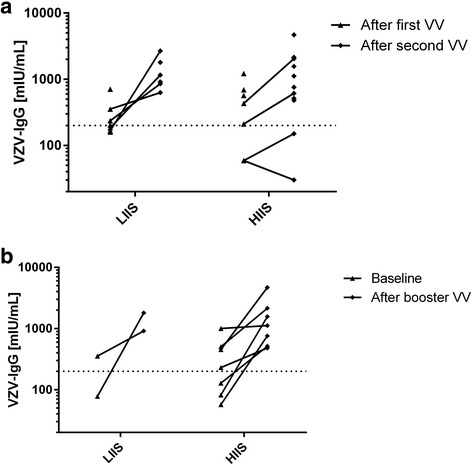
Assessment of immune response. **a** VZV-IgG levels after first and/or second dose of the varicella vaccine. For patients who received both a first and second varicella vaccination within the study, the longitudinal response is indicated by horizontal connecting lines. Patients who received either only a first dose of the varicella vaccine or a booster dose are indicated by lack of horizontal connecting lines. Baseline levels in patients only having received a booster dose are not shown. **b** VZV-IgG levels for those patients having received only a booster dose, including the baseline levels

Among patients who had received a single dose of the VZV vaccine before start of IS, the VZV-IgG baseline levels were 230 (57-1003) mIU/ml (median (range)). Figure 2 shows post-vaccination titers. Following the first VZV vaccination within this study, the median VZV-IgG level across all patients was 224 (59-1219) mIU/ml (LIIS subgroup 203 (159-707) mIU/ml, HIIS subgroup 430 (59-1219) mIU/ml). After the second vaccination, VZV-IgG levels increased to 882 (30-4685) mIU/ml (LIIS group 1035 (627-2671) mIU/ml, HIIS group 684 (30-4685) mIU/mL). The difference in the VZV-IgG levels between the LIIS and the HIIS groups was not significant (Mann-Whitney test *p*=0.67 after first vaccination, *p*=0.26 after second vaccination). Similarly, the absolute (Δ) and the relative (fold-) increase in VZV-IgG after first to after second vaccination (LIIS Δ796 mIU/ml and 4.9-fold, HIIS Δ393 mIU/ml and 3.5-fold, respectively) were not significantly different between these groups (Mann-Whitney test *p*=0.26 and *p*=0.49, respectively). Among participants only receiving a booster vaccination, all achieved VZV-IgG levels >500 mIU/ml, i.e. 1117 (513-4685) mIU/ml. Two participants in the HIIS group did not achieve an increase in VZV-IgG >200 mIU/ml despite two vaccinations. Patient 1 (on MMF monotherapy) and patient 2 (on leflunomide and abatacept therapy) both had VZV-IgG ELISA values in the negative range of the assays.

### Safety assessments

The VZV vaccinations were generally well tolerated. There were only minor adverse events, Table 3. There were no cases of rash or other vaccine-induced varicella disease symptoms and no evidence of PRD flare following vaccination.

**Table 3:** Adverse events within four weeks after varicella vaccination

Adverse event	LIIS group	HIIS group
Local reaction at injection site – n (%)	1 (11%)	1 (7%)
Systemic rash	0 (0%)	0 (0%)
Elevated temperature	0 (0%)	1 (7%)
Headache	0 (0%)	1 (7%)
Vomiting/gastroenteritis	0 (0%)	1 (7%)
Arthralgia/joint complaints^a^	3 (33%)	1 (7%)

*Abbreviations: HIIS* high-intensity immunosuppression, *LIIS* low-intensity immunosuppression.

^a^Transient use of nonsteroidal anti-inflammatory drugs.

### Efficacy following vaccination

After a median follow-up of three years, no patient had suffered from breakthrough chickenpox or from HZ. A known exposure to VZV was documented in three cases (one to herpes zoster and two to chickenpox).

## Discussion

To our knowledge, this is the first study employing a pre-vaccination stratifying algorithm centered on immunologic criteria, rather than on type of IS medication used, to decide whether a IS patient got vaccinated.

The implementation of the pre-vaccination checklist ensured immune competence prior to vaccination. On that basis we demonstrate the safety and immunogenicity of VZV vaccination in an insufficiently protected cohort of patients with PRD receiving LIIS or HIIS, including biologic DMARDs.

All patients receiving LIIS and/or only a booster vaccination developed an increase in VZV-IgG above 200 mIU/ml (defined as positive by the manufacturer of the assay). Two out of 14 patients receiving HIIS did not: one on MMF (which has anti-proliferative activity in B- and T-cells) and one on leflunomide and abatacept (an agent blocking T cell co-stimulation). Abatacept and MMF have previously been associated with poor responses to non-live vaccine immunization [21–23]. Our study supports previous studies on VZV vaccination in patients with PRD receiving synthetic DMARD and a small cohort on biological agents in which a low rate of flares and rash were demonstrated, even though immunogenicity has been lower in these studies [15, 16, 24, 25].

For the two-step checklist, we selected parameters regarding humoral and cellular immunity that in previous studies indicated the ability to control the standard-dose Oka virus strain of the VZV vaccine even during severe IS [26–29]. We emphasize that testing T cell function may be simplified using a standard TB-IGRA, such as the widely available QuantiFERON-TB Gold® and the TB-EliSpot® tests which both include a mitogen-induced T cell proliferation (to *phytohemagglutinin, PHA*) as positive control.

In our opinion, the decision to administer both (first and booster) live vaccination might be better based on the actual immunoreactivity rather than on the type of IS medication.

The alternative is to delay the initiation of IS, use a low-dose glucocorticoid bridging therapy or interrupt IS for several weeks [17, 27]. In our experience, the latter approach is often refused both by treating physicians and patients due to an increased risk of disease flares. An intermediate solution would be to administer the first dose of the VZV vaccine prior to IS, and the second dose while on IS to optimize safety and immune response while decreasing the risk of disease flare.

In 2009, Germany initiated a general 2-dose VZV vaccine schedule for healthy children receiving the first dose between the age of 11 to 14 months and the second dose between the age of 15 to 23 months [30, 31]. General VZV vaccination is recommended in 36 countries, whereas an additional 9 countries only pursue a targeted vaccination regimen of populations at risk (Additional file 1: Table S1) [32–34]. Nonetheless, in juvenile idiopathic arthritis (JIA), the most common PRD, the peak age of onset is the second year of life, i.e. IS is often required before VZV immunization has been completed [35]. We demonstrate a rather high proportion of pre-existing VZV protection in our large cohort of around 2800 patients; 94% of screened patients were considered immune to VZV based on history of appropriate vaccination (92.1%) or chickenpox. This compares favorably to published national data on VZV vaccination coverage in Germany (around 70%) [36]. One reason may be that in our center a strong emphasis is placed on the recommendation to catch-up on vaccinations prior to initiating IS if possible.

In countries with targeted immunization strategies the pre-vaccination checklist may be even more helpful protecting susceptible patients with urgent need for IS and high risk of chickenpox exposure. In patients without a 2-dose VZV vaccination and uncertain history of VZV disease, testing for VZV-IgG seems advisable prior to long-term IS, and negative or borderline VZV-IgG levels should lead to vaccination. On the other hand, post-exposure prophylaxis with acyclovir and VZV hyperimmune globulin may fail in preventing varicella disease during IS [1, 37–39]. Furthermore, reactivation and HZ often cause substantial morbidity during IS and occur much more often from latent wild-virus infection than after reactivation of the latent VZV vaccine strain [40–43].

One limitation of our study is that this is a rather small cohort of patients treated with various combinations of IS. We only documented an increase in VZV-IgG but CMI may be just as important. Long-term data for VZV-IgG and cell-mediated immunity (CMI) in patients with leukemia or immunosuppression (IS) who received two doses of the VZV vaccine showed that VZV-IgG peaks and then declines over time, whereas long-lasting VZV-CMI persists and protects between 75-100% of vaccinees from clinically relevant VZV disease after exposure [25, 39, 40, 41, 44, 45]. Furthermore, post-vaccination titers are less in magnitude when compared to that following wild type VZV infection [25, 40, 39]. In a cohort of 20 patients receiving HIIS at our center, including rituximab, all patients with a history of chickenpox maintained very high seroconversion levels of VZV-IgG (range 1052 to greater than 2000 mIU/ml) (unpublished data).

It is noteworthy that none of the screened patients “failed” the stratification process. Based on further experience and data the pre-vaccination checklist may be simplified in the future, especially concerning booster-live vaccinations. Moreover, the application of the pre-vaccination checklist for first measles-mumps-rubella (MMR) vaccination on LIIS and booster MMR vaccination on LIIS and HIIS should be discussed. Two minor modifications have been proposed by experts regarding the application of the MMR vaccine: first, proof of an increase in antibody titer following “diagnostic immunization” with inactivated vaccines and, second, a higher threshold for CD4 + T cells (>500/μl) [46].

## Conclusions

In summary, we demonstrated that the monovalent VZV vaccine was well-tolerated and mostly efficacious in a cohort of VZV-susceptible patients with PRD receiving ongoing IS (including biologic DMARDs) after proving immunologic reactivity by means of a simple checklist. We believe that the safety and efficacy of this easy-to-obtain approach should be prospectively evaluated in a larger patient cohort.

## Additional file

Additional file 1: List of 45 countries with mandatory varicella zoster virus vaccination (according to the national vaccination schedule) retrieved from the WHO vaccine-preventable diseases monitoring system 2017*. (DOCX 19 kb)

## Abbreviations

CMI: cell-mediated immunity
DMARD: disease-modifying antirheumatic drug
EULAR: European League Against Rheumatism
HIIS: high-intensity immunosuppression
Ig: immune globulin
IGRA: interferon-gamma release assay
IS: immunosuppression
IVIG: intravenous immune globulins
JIA: juvenile idiopathic arthritis
LIIS: low-intensity immunosuppression
MMF: mycophenolate mofetil
MMR: measles, mumps, rubella
PRD: pediatric rheumatic diseases
STIKO: Ständige Impfkommission (German Standing Committee On Vaccination)
VZV: varizella zoster virus
WBC: white blood cells

## References

[1] Wiegering V, Schick J, Beer M, Weissbrich B, Gattenlohner S, Girschick HJ, Liese J, Schlegel PG, Eyrich M (2011). Varicella-zoster virus infections in immunocompromised patients - a single centre 6-years analysis. BMC pediatrics.

[2] Ha K, Baba K, Ikeda T, Nishida M, Yabuuchi H, Takahashi M (1980). Application of live varicella vaccine to children with acute leukemia or other malignancies without suspension of anticancer therapy. Pediatrics.

[3] Takahashi M (1986). Clinical overview of varicella vaccine: development and early studies. Pediatrics.

[4] Chaves Tdo S, Lopes MH, de Souza VA, Dos Santos SS, Pereira LM, Reis AD, David-Neto E (2005). Seroprevalence of antibodies against varicella-zoster virus and response to the varicella vaccine in pediatric renal transplant patients. Pediatr. Transplant.

[5] Furth SL, Arbus GS, Hogg R, Tarver J, Chan C, Fivush BA (2003). Southwest Pediatric Nephrology Study G: Varicella vaccination in children with nephrotic syndrome: a report of the Southwest Pediatric Nephrology Study Group. J. Pediatr.

[6] Gershon AA, Steinberg SP, Gelb L (1986). Live attenuated varicella vaccine use in immunocompromised children and adults. Pediatrics.

[7] Kano H, Mizuta K, Sakakihara Y, Kato H, Miki Y, Shibuya N, Saito M, Narita M, Kawarasaki H, Igarashi T (2002). Efficacy and safety of immunization for pre- and post- liver transplant children. Transplantation.

[8] Lu Y, Bousvaros A (2010). Varicella vaccination in children with inflammatory bowel disease receiving immunosuppressive therapy. J. Pediatr. Gastroenterol. Nutr.

[9] Weinberg A, Horslen SP, Kaufman SS, Jesser R, Devoll-Zabrocki A, Fleckten BL, Kochanowicz S, Seipel KR, Levin MJ (2006). Safety and immunogenicity of varicella-zoster virus vaccine in pediatric liver and intestine transplant recipients. American journal of transplantation : official journal of the American Society of Transplantation and the American Society of Transplant Surgeons.

[10] Zamora I, Simon JM, Da Silva ME, Piqueras AI (1994). Attenuated varicella virus vaccine in children with renal transplants. Pediatr. Nephrol.

[11] Ghaffar F, Carrick K, Rogers BB, Margraf LR, Krisher K, Ramilo O (2000). Disseminated infection with varicella-zoster virus vaccine strain presenting as hepatitis in a child with adenosine deaminase deficiency. Pediatr. Infect. Dis. J.

[12] Kramer JM, LaRussa P, Tsai WC, Carney P, Leber SM, Gahagan S, Steinberg S, Blackwood RA (2001). Disseminated vaccine strain varicella as the acquired immunodeficiency syndrome-defining illness in a previously undiagnosed child. Pediatrics.

[13] Gershon AA, LaRussa P, Steinberg S (1996). The varicella vaccine. Clinical trials in immunocompromised individuals. Infectious disease clinics of North America.

[14] Barbosa CM, Terreri MT, Rosario PO, de Moraes-Pinto MI, Silva CA, Hilario MO (2012). Immune response and tolerability of varicella vaccine in children and adolescents with systemic lupus erythematosus previously exposed to varicella-zoster virus. Clin. Exp. Rheumatol.

[15] Pileggi GS, de Souza CB, Ferriani VP (2010). Safety and immunogenicity of varicella vaccine in patients with juvenile rheumatic diseases receiving methotrexate and corticosteroids. Arthritis care & research.

[16] Toplak N, Avcin T (2015). Long-term safety and efficacy of varicella vaccination in children with juvenile idiopathic arthritis treated with biologic therapy. Vaccine.

[17] Broyer M, Tete MJ, Guest G, Gagnadoux MF, Rouzioux C (1997). Varicella and zoster in children after kidney transplantation: long-term results of vaccination. Pediatrics.

[18] Kitai IC, King S, Gafni A (1993). An economic evaluation of varicella vaccine for pediatric liver and kidney transplant recipients. Clinical infectious diseases : an official publication of the Infectious Diseases Society of America.

[19] Levin MJ (2008). Varicella vaccination of immunocompromised children. Am. J. Infect. Dis.

[20] Sartori AM (2004). A review of the varicella vaccine in immunocompromised individuals. International journal of infectious diseases : IJID : official publication of the International Society for Infectious Diseases.

[21] Heijstek MW, Ott de Bruin LM, Bijl M, Borrow R, van der Klis F, Kone-Paut I, Fasth A, Minden K, Ravelli A, Abinun M (2011). EULAR recommendations for vaccination in paediatric patients with rheumatic diseases. Ann. Rheum. Dis.

[22] Wallace CA, Ruperto N, Giannini E, Childhood A, Rheumatology Research A (2004). Pediatric Rheumatology International Trials O, Pediatric Rheumatology Collaborative Study G: Preliminary criteria for clinical remission for select categories of juvenile idiopathic arthritis. J. Rheumatol.

[23] Rubin LG, Levin MJ, Ljungman P, Davies EG, Avery R, Tomblyn M, Bousvaros A, Dhanireddy S, Sung L, Keyserling H (2014). 2013 IDSA clinical practice guideline for vaccination of the immunocompromised host. Clinical infectious diseases : an official publication of the Infectious Diseases Society of America.

[24] Di Genova G, Savelyeva N, Suchacki A, Thirdborough SM, Stevenson FK (2010). Bystander stimulation of activated CD4+ T cells of unrelated specificity following a booster vaccination with tetanus toxoid. Eur. J. Immunol.

[25] Crnkic Kapetanovic M, Saxne T, Jonsson G, Truedsson L, Geborek P (2013). Rituximab and abatacept but not tocilizumab impair antibody response to pneumococcal conjugate vaccine in patients with rheumatoid arthritis. Arthritis research & therapy.

[26] Ribeiro AC, Laurindo IM, Guedes LK, Saad CG, Moraes JC, Silva CA, Bonfa E (2013). Abatacept and reduced immune response to pandemic 2009 influenza A/H1N1 vaccination in patients with rheumatoid arthritis. Arthritis care & research.

[27] Karbasi-Afshar R, Izadi M, Fazel M, Khedmat H (2015). Response of transplant recipients to influenza vaccination based on type of immunosuppression: A meta-analysis. Saudi J Kidney Dis Transpl.

[28] Hardy I, Gershon AA, Steinberg SP, LaRussa P (1991). The incidence of zoster after immunization with live attenuated varicella vaccine. A study in children with leukemia. Varicella Vaccine Collaborative Study Group. N. Engl. J. Med.

[29] Bergen RE, Diaz PS, Arvin AM (1990). The immunogenicity of the Oka/Merck varicella vaccine in relation to infectious varicella-zoster virus and relative viral antigen content. J. Infect. Dis.

[30] Diaz PS, Smith S, Hunter E, Arvin AM (1989). T lymphocyte cytotoxicity with natural varicella-zoster virus infection and after immunization with live attenuated varicella vaccine. J. Immunol.

[31] White CJ (1997). Varicella-zoster virus vaccine. Clinical infectious diseases : an official publication of the Infectious Diseases Society of America.

[32] Zhang Y, Cosyns M, Levin MJ, Hayward AR (1994). Cytokine production in varicella zoster virus-stimulated limiting dilution lymphocyte cultures. Clin. Exp. Immunol.

[33] Koch-Institut R (2004). Begründung der STIKO für eine allgemeine Varizellenimpfung. Epidemiologisches Bulletin.

[34] Koch-Institut R (2009). Impfung gegen Varizellen im Kindesalter: Empfehlung einer zweiten Varizellenimpfung Empfehlung und Begründung. Epidemiologisches Bulletin.

[35] WHO vaccine-preventable diseases: monitoring system. 2017 global summary [http://apps.who.int/immunization_monitoring/globalsummary]

[36] Control ECfDPa: Varicella vaccination in the European Union. 2015.

[37] Macartney KK, Burgess MA (2008). Varicella vaccination in Australia and New Zealand. J. Infect. Dis.

[38] Sullivan DB, Cassidy JT, Petty RE (1975). Pathogenic implications of age of onset in juvenile rheumatoid arthritis. Arthritis and rheumatism.

[39] Rieck T, Feig M, Eckmanns T, Benzler J, Siedler A, Wichmann O (2014). Vaccination coverage among children in Germany estimated by analysis of health insurance claims data. Human vaccines & immunotherapeutics.

[40] Feldman S, Lott L (1987). Varicella in children with cancer: impact of antiviral therapy and prophylaxis. Pediatrics.

[41] Kumagai T, Chiba Y, Fujiwara M, Hanazono H, Nakamura S, Chiba S, Nakao T, Takahashi M (1980). Humoral and cellular immune response to varicella-zoster virus in children inoculated with live attenuated varicella vaccine. Biken journal.

[42] Harpaz R, Ortega-Sanchez IR, Seward JF, Advisory Committee on Immunization Practices Centers for Disease C, Prevention: Prevention of herpes zoster: recommendations of the Advisory Committee on Immunization Practices (ACIP). MMWR Recommendations and reports:Morbidity and mortality weekly report Recommendations and reports/Centers for Disease Control 2008, 57(RR-5):1-30; quiz CE32-34. 18528318

[43] Smitten AL, Choi HK, Hochberg MC, Suissa S, Simon TA, Testa MA, Chan KA (2007). The risk of herpes zoster in patients with rheumatoid arthritis in the United States and the United Kingdom. Arthritis and rheumatism.

[44] Gershon AA (1995). Varicella-zoster virus: prospects for control. Advances in pediatric infectious diseases.

[45] Oitani K (1999). Expression of interleukin-2 receptor, CD25, on CD4 lymphocytes in response to varicella-zoster virus antigen among patients with malignancies immunized with live attenuated varicella vaccine. Pediatrics international : official journal of the Japan Pediatric Society.

[46] Speth F, Haas JP, Kneitz C, Warnatz K, Minden K: [Vaccinations in immunosuppressed young rheumatics in transition]. arthritis + rheuma 2017, 37(1):39-58.

