# Supplementary material for: Varicella-zoster-virus vaccination in immunosuppressed children with rheumatic diseases using a pre-vaccination check list
Source: Pediatr Rheumatol Online J. 2018 Mar 2;16:15. doi: 10.1186/s12969-018-0231-3 (PMC5833060; doi:10.1186/s12969-018-0231-3)
Supplement: Supplementary file 1 — List of 45 countries with mandatory varicella zoster virus vaccination (according to the national vaccination schedule) retrieved from the WHO vaccine-preventable diseases monitoring system 2017*. (DOCX 19 kb) [file 12969_2018_231_MOESM1_ESM.docx]

Additional file 1: Table S1: List of 45 countries with mandatory varicella zoster virus vaccination (according to the national vaccination schedule) retrieved from the WHO vaccine-preventable diseases monitoring system 2017*

| Country | number of immunizations | Age | Entire country? | Comment |
| --- | --- | --- | --- | --- |
| Andorra | 2 | 15 mo, 3 yrs | yes |  |
| Antigua | 1 | 3-5 yrs | no |  |
| Argentina | 1 | 15 mo, 3 yrs | yes |  |
| Australia | 1 | 10-15 yrs | yes | Zoster vaccine at 70 yrs |
| Bahamas | 2 | 1 yrs, 4-5 yrs | yes |  |
| Bahrain | 2 | 1 yr, 3 yrs | yes |  |
| Barbados | 1 | 1 yr | yes |  |
| Brazil | 1 | 15 mo | yes |  |
| Canada | 2 | 12 mo, 18 mo-6 yrs | yes | Zoster vaccine at 65-70 yrs (Ontario only) |
| Colombia | 1 | 1 yr | yes |  |
| Costa Rica | 1 | 15 mo | yes |  |
| Cyprus | 2 | 13-18 mos, 4-6 yrs | yes |  |
| Ecuador | 1 | 15 mos | yes |  |
| Finland | 2 | 12 mos, 6 yrs | yes |  |
| France | N/A | N/A | N/A | Zoster vaccine at 65-74 yrs |
| Germany | 2 | 11-14 mos, 15-23mos | yes |  |
| Greece | 2 | 12-15 mos, 4-6 yrs | yes |  |
| Grenada | 1 | 12 mos | yes |  |
| Guyana | N/A | N/A | no | VZV vaccination at first contact + 6 wks later |
| Iran | N/A | N/A | yes | VZV vaccination in children with lymphatic and hematologic malignancies and their siblings |
| Israel | N/A | N/A | yes | VZV vaccination in certain risk groups |
| Italy | 2 | 13-15 mos, 5-6 yrs | yes |  |
| Japan | 2 | 12 mos, 18 mos | yes |  |
| Kuwait | N/A | N/A | no | VZV vaccination in HCW at risk |
| Latvia | 2 | 12-15 mos, 7 yrs | yes | second VZV dose from January 2019 |
| Mexico | 1 | 1 yr | yes |  |
| New Zealand | N/A | N/A | yes | VZV vaccination in eligible individuals |
| Oman | 1 | 12 mos | yes |  |
| Panama | 2 | 15 mos, 4 yrs | yes |  |
| Paraguay | 1 | 15 mos | yes |  |
| Qatar | 2 | 12 mos, 4-6 yrs | yes |  |
| Republic of (South) Korea | 1 | 12-15 mos | yes |  |
| Russian Federation | 1 | 12 mos | no |  |
| Saint Lucia | N/A | N/A | yes | VZV vaccination in high-risk individuals |
| San Marino | 2 | 15 mos, 10 yrs | yes |  |
| Saudi Arabia | 2 | 18 mos, 6 yrs | yes |  |
| Slovenia | N/A | N/A | yes | VZV vaccination in high-risk individuals |
| Spain | 2 | 15 mos, 3-4 yrs | yes |  |
| Switzerland | 1 | 11-15 yrs | yes |  |
| Trinidad & Tobago | 1 | 12 mos | yes |  |
| Turkey | 1 | 12 mos | yes |  |
| United Arab Emirates | 2 | 12 mos, 5-6 yrs | yes |  |
| United Kingdom | N/A | N/A | yes | VZV vaccination in non-immune HCW |
| United States of America | 2 | 12 mos, 4 yrs | yes | Zoster vaccine at 60 yrs |
| Uruguay | 2 | 1 yr, 5 yrs | yes |  |
| Abbreviations: HCW, health care workers; mos, months; N/A, not applicable; VZV, varicella zoster virus; yr, year; yrs, years  * http://apps.who.int/immunization_monitoring/globalsummary | | | | |
